# Editorial: Molecular and cellular mechanisms of bone remodeling

**DOI:** 10.3389/fendo.2026.1898761

**Published:** 2026-06-30

**Authors:** Sandeep Kumar, Antonio Desmond McCarthy, Claudia Sedlinsky

**Affiliations:** 1 Department of Microbiology/Immunology, Tulane University, New Orleans, LA, United States; 2 Osteopathy and Mineral Metabolism Research Laboratory (LIOMM), Facultad de Ciencias Exactas, Universidad Nacional de La Plata, La Plata, Argentina; 3 Endocrinology Unit, Hospital Cesar Milstein, Buenos Aires, Argentina

**Keywords:** bone, muscle, osteocyte, osteoporosis, sarcopenia

## Introduction

**Figure 1:**
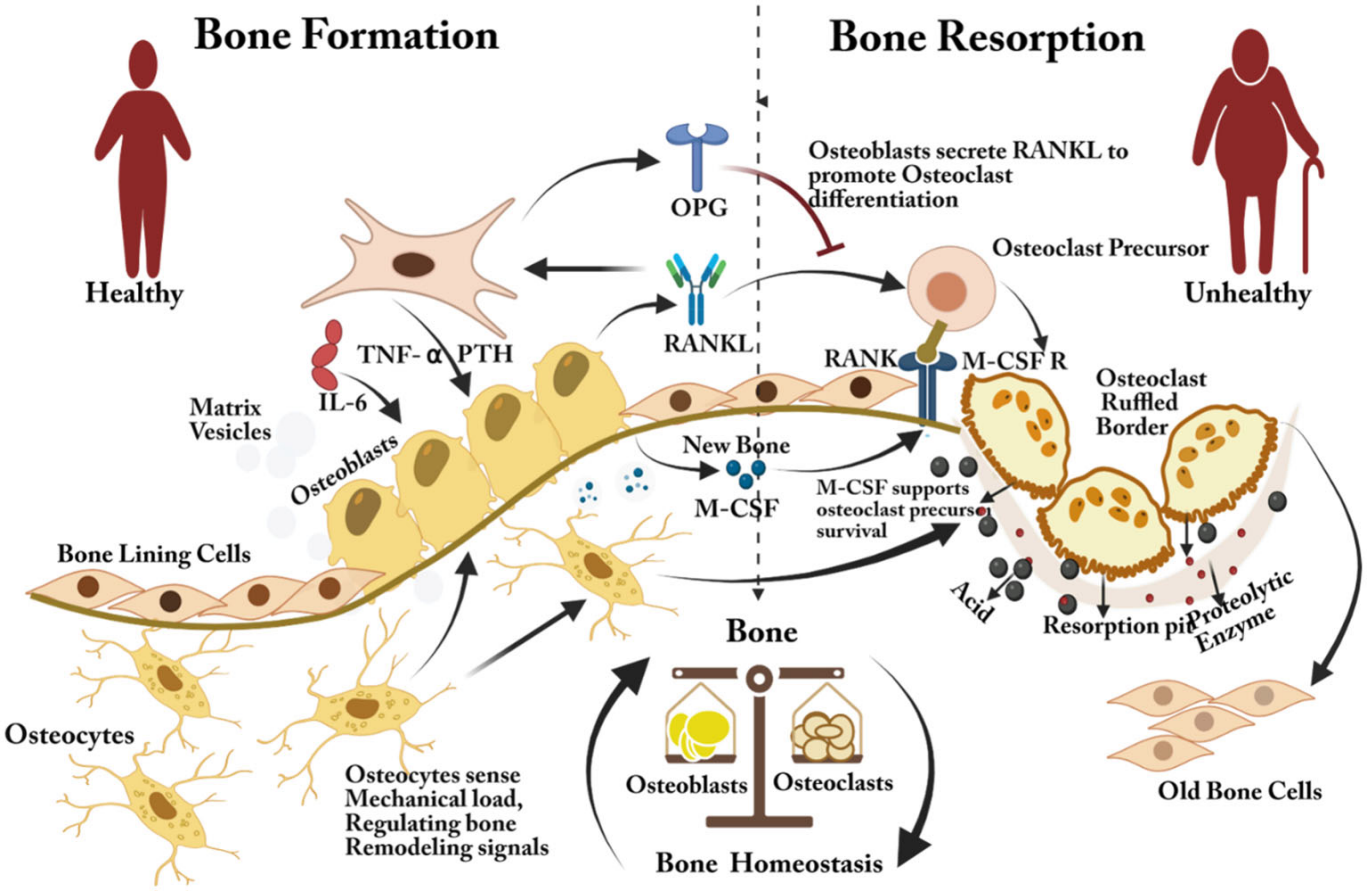
Schematic representation of bone remodeling showing the coordinated activities of osteocytes, osteoblasts and osteoclasts. Osteoblast-derived OPG and RANKL regulate osteoclast differentiation through the RANK/RANKL/OPG axis, while osteocytes sense mechanical stimuli and coordinate bone formation and resorption to maintain bone homeostasis.

Bone remodeling is a critical physiological process that maintains skeletal integrity, repairs micro-damage, and regulates mineral homeostasis, relying on the coordinated actions of bone-resorbing osteoclasts, bone-forming osteoblasts, and osteocytes serving as mechanosensors and local remodeling coordinators (1–3). Disruptions in this balance can lead to disorders such as osteoporosis, osteopetrosis, and Paget’s disease (**Figure 1**) (4–6). Recent research has revealed new layers of complexity in the regulation of bone remodeling, including new signaling pathways, intracellular mechanisms, epigenetic regulation, and cross-communication with immune and endocrine systems.

Traditionally viewed as a balance between bone resorption and formation, bone remodeling is now understood as a dynamic process influenced by immune cells, metabolic signaling, stem cell niches, microbiota-derived factors and local microenvironmental cues. The field of osteo-immunology highlights bone as an active immunometabolic tissue vital for maintaining homeostasis (7).

Osteocytes play a central role in this network, facilitating communication among osteoclasts, osteoblasts, endothelial and immune cells, through the secretion of RANKL, sclerostin, and inflammatory mediators, which enable rapid bone adaptation to mechanical and physiological stresses. Increasing evidence suggests that osteocyte dysfunction is a central driver of osteoporosis, osteoarthritis and age-related skeletal fragility (6, 8).

Additionally, immune cells such as T and B cells, dendritic cells and macrophages, significantly influence osteoclast formation via cytokine secretion, leading to a view of bone loss as an immunological disorder as well as a skeletal disease. The concept of “immunoporosis” has emerged to describe bone loss associated with chronic low-grade inflammation due to aging, obesity, autoimmune disease and metabolic dysfunction (9, 10).

Mesenchymal and hematopoietic stem cells, endothelial cells, and other bone marrow stromal populations, create specialized niches that coordinate bone regeneration and hematopoiesis; and its alterations lead to impaired fracture healing, osteoporosis, malignancy-associated bone disease, and age-related skeletal decline (11). Recent studies highlight metabolic factors, such as cellular metabolism and mitochondrial function, as critical in regulating osteoblast and osteoclast activity, emphasizing the profound impact of bone microenvironment on skeletal health (12, 13).

Our current Research Topic encompasses a significant body of studies that underscore the multifaceted nature of bone health, highlighting the intricate interplay between hormonal, metabolic, immune, and environmental factors in bone remodeling and associated conditions. The contributions reflect a diverse array of themes that converge on understanding bone physiology, pathology, and therapeutic potential across demographics and conditions.

## Hormonal, metabolic and immune regulation of bone metabolism

Hormonal and metabolic factors are recurring themes across the studies, emphasizing their critical roles in bone health. For instance, the work by Brona et al. delineates how pregnancy-related hormonal shifts regulate calcium homeostasis. Yan et al. highlight the multiple factors that influence the development of osteoporosis, including hormonal regulation, and metabolic and immunoinflammatory imbalance. These findings resonate with the exploration by Yang et al. of how systemic inflammation and hormonal dysregulation in asthma can detrimentally affect bone health. Similarly, Li et al. have demonstrated that metabolic interventions such as inhibition of AMPK may reverse the bone pathologic phenotype of Gnathodiaphyseal Dysplasia, a rare genetic disease caused by Anoctamin 5 mutations characterized by increased osteogenesis and reduced osteoclastogenesis.

## Regulatory pathways affecting bone cells

Gao et al. report the emerging role of chemokine signaling networks as critical regulators of bone remodeling, that interact with major bone signaling cascades to coordinate cellular communication within bone microenvironment; while Wu et al. demonstrate that lactoferrin, an endogenous multifunctional glycoprotein, exerts a strong positive regulatory effect on bone metabolism through both direct and indirect mechanisms to improve bone integrity. Liu et al. confirm that Piezo1 is a key mechanosensitive ion channel that regulates bone homeostasis by converting mechanical stimuli into Ca^2+^-dependent signaling in osteocytes, osteoblasts and bone marrow stromal cells (BMSC), and describe its decline with aging as part of the pathophysiology of osteoporosis. Li et al. describe integrin α5β1 as a promotor of bone formation; however, in pathological conditions such as osteoporosis, osteoarthritis and bone metastasis, dysregulated α5β1 signaling can contribute to impaired bone formation and excessive resorption. On the other hand, Bakari Murusuri and Tang describe the role of ferroptosis, an iron-dependent form of cell death, as a contributor to the pathogenesis of osteoporosis, and how factors such as aging, estrogen deficiency, diabetes, glucocorticoid exposure, and obesity further exacerbate ferroptosis in bone cells.

## Regenerative mechanisms and therapeutic modalities

Bikle‘s work on fracture repair describes the multistage nature of fracture healing, and the mechanisms of action of the anabolic agents teriparatide and abaloparatide through their interaction with PTH receptor that enhance fracture healing through activation of signaling pathways such as cAMP/PKA, Wnt, BMP, IGF-1, and Ephrin B2/EphB4. On the other hand, Qiao et al. demonstrate that inhibition of histone methyltransferase G9a by the therapeutic agent UNC0638 promotes the osteogenic potential of BMSC, thus reversing a diabetic bone phenotype.

## Endocrine regulation and metabolic crosstalk

Emerging research, presented by Anees et al., illustrates how osteocytes are increasingly recognized as endocrine regulators that influence systemic metabolism beyond their traditional role in skeletal maintenance. The evidence presented suggests that osteocyte-derived signals can modulate adipocyte browning and thermogenic gene expression across adipose depots. However, the evidence remains preliminary and further research is needed to establish the physiological relevance of osteocyte-mediated adipocyte browning, and its therapeutic potential in metabolic and skeletal disorders.

In summary, the articles of this research topic collectively reveal the complex and integrative nature of bone health, yet they highlight the necessity for a more critical perspective on the translational implications of their findings. Future research should aim to bridge current knowledge gaps through comprehensive, population-based studies that consider the intricate interplays among hormones, inflammation, and other systemic factors.

## Future directions

The next decade of bone remodeling research is poised for a transformative paradigm shift, evolving from a narrow focus on isolated cellular pathways to a comprehensive understanding of the skeleton as a dynamic, integrated immune metabolic and mechanoresponsive organ system. Emerging evidence underscores that bone homeostasis is not merely a product of osteocyte, osteoclast, and osteoblast activity, but rather the result of intricate interactions involving diverse cellular entities including immune cells, mesenchymal stem cells, vascular networks, and endocrine signals. These overlapping systems are interconnected through complex metabolic pathways that govern skeletal integrity, highlighting the need for future research to elucidate these multidimensional regulatory networks rather than individual molecular targets.

A priority moving forward will be the application of cutting-edge technologies -such as single-cell multiomics, spatial transcriptomics, proteomics, and artificial intelligence-assisted systems biology - to dissect the cellular heterogeneity within the bone microenvironment. By leveraging these innovative methodologies, researchers will be able to identify previously unrecognized subsets of osteoblasts, osteoclasts, osteocytes, immune cells, and supportive stroma that contribute to bone remodeling under both physiological and pathological conditions. This comprehensive cellular mapping will aid in biomarker discovery, driving the development of precision medicine strategies tailored for osteoporosis and inflammatory bone disorders.

Looking ahead, the future of bone remodeling research lies at the intersection of osteoimmunology, systems biology, and precision medicine. Emerging therapeutic strategies are likely to transcend traditional antiresorptive and anabolic agents, incorporating targeting of immune pathways, macrophage polarization, stem-cell communication networks, and microenvironmental regulators. These holistic approaches have the potential to revolutionize the management of a spectrum of skeletal conditions, including osteoporosis, inflammatory arthritis, fracture nonunion, bone metastasis, and age-associated skeletal degeneration.

In addition to the advancements in osteoimmunology, researchers should critically investigate how macrophage polarization, dendritic cell activation, T-cell subsets, and cytokine networks intricately regulate skeletal homeostasis. Unlocking these immuno-skeletal interactions may pave the way for innovative immunomodulatory therapies that may not only suppress pathological bone resorption, but also promote bone regeneration. Moreover, a deeper focus on cellular metabolism and mitochondrial functionality in bone remodeling is warranted, as metabolic reprogramming, mitochondrial transfer, oxidative stress, and nutrient-sensing pathways are being recognized as pivotal regulators of osteoblast and osteoclast differentiation.

Furthermore, recent discoveries related to regulated cell death mechanisms -such as ferroptosis, pyroptosis, cuproptosis, and PANoptosis - are offering new insights into skeletal pathology. Investigating how these varied cell-death programs influence bone cell survival, remodeling dynamics, and tissue regeneration, could unveil novel therapeutic targets. From a translational standpoint, the refinement of osteocyte-targeted therapies, evolution of stem cell-based regenerative techniques, and advancements in biomaterial-assisted bone repair, extracellular vesicle therapeutics, and gene-editing technologies, are set to revolutionize the treatment landscape for skeletal diseases. Coupling these innovative strategies with precision diagnostics and predictive biomarkers will enable the development of personalized interventions, specifically tailored to individual remodeling profiles.

## Concluding remarks

Bone remodeling is evolving beyond a simplistic balance between osteoclast-mediated bone resorption and osteoblast-mediated formation, into a complex, highly coordinated, and dynamic process influenced by an intricate tapestry of cellular interactions throughout the skeletal system. Advances in the areas of osteoimmunology, mechanobiology, stem cell biology, and precision medicine are fundamentally reshaping our understanding of skeletal homeostasis and the pathogenesis of disease. Despite the substantial progress achieved, critical challenges remain in deciphering the spatial and temporal complexities of cellular communication within the bone microenvironment. Bridging these gaps in knowledge will need multidisciplinary approaches that integrate molecular biology, computational sciences, bioengineering, and clinical investigation.

The convergence of multiomics technologies, artificial intelligence, and regenerative medicine presents unprecedented avenues to uncover novel therapeutic targets and predictive biomarkers. Ultimately, the forthcoming advances in bone remodeling research are anticipated to shift the focus from mere symptom management towards precision-based prevention, restoration of skeletal homeostasis, and promotion of bone regeneration. Such innovations hold the promise to not only revolutionize treatments for osteoporosis and inflammatory bone diseases, but also redefine our understanding of healthy aging and musculoskeletal longevity. As the realms of osteoimmunology and immunometabolism continue to advance, the skeleton is emerging as a critical regulator of whole-body physiology, solidifying the position of bone remodeling research at the cutting edge of translational and precision medicine.

## Editorial take-home message

The greatest challenge in bone remodeling research is no longer identifying the major cellular players, but understanding how skeletal, immune, vascular and metabolic networks interact across space and time. Bridging these knowledge gaps will be crucial for developing next-generation therapies that not only prevent bone loss but also regenerate functional bone and promote healthy aging.
